# A SARS-CoV-2 coronavirus nucleocapsid protein antigen-detecting lateral flow assay

**DOI:** 10.1371/journal.pone.0258819

**Published:** 2021-11-10

**Authors:** Benjamin D. Grant, Caitlin E. Anderson, Luis F. Alonzo, Spencer H. Garing, John R. Williford, Ted A. Baughman, Rafael Rivera, Veronika A. Glukhova, David S. Boyle, Puneet K. Dewan, Bernhard H. Weigl, Kevin P. Nichols

**Affiliations:** 1 Global Health Labs, Bellevue, Washington, United States of America; 2 Intellectual Ventures Lab, Bellevue, Washington, United States of America; 3 PATH, Seattle, Washington, United States of America; Waseda University: Waseda Daigaku, JAPAN

## Abstract

Inexpensive, simple, rapid diagnostics are necessary for efficient detection, treatment, and mitigation of COVID-19. Assays for SARS-CoV2 using reverse transcription polymerase chain reaction (RT-PCR) offer good sensitivity and excellent specificity, but are expensive, slowed by transport to centralized testing laboratories, and often unavailable. Antigen-based assays are inexpensive and can be rapidly mass-produced and deployed at point-of-care, with lateral flow assays (LFAs) being the most common format. While various manufacturers have produced commercially available SARS-Cov2 antigen LFAs, access to validated tests remains difficult or cost prohibitive in low-and middle-income countries. Herein, we present a visually read open-access LFA (OA-LFA) using commercially-available antibodies and materials for the detection of SARS-CoV-2. The LFA yielded a Limit of Detection (LOD) of 4 TCID_50_/swab of gamma irradiated SARS-CoV-2 virus, meeting the acceptable analytical sensitivity outlined by in World Health Organization target product profile. The open-source architecture presented in this manuscript provides a template for manufacturers around the globe to rapidly design a SARS-CoV2 antigen test.

## Introduction

The novel coronavirus, SARS-CoV-2, was identified in China in late 2019 and is characterized by significant mortality, morbidity, and infectiousness [1]. As of May 2021, COVID-19, the disease caused by SARS-CoV-2, has infected 216 million people worldwide and caused nearly 4.5 million deaths [2].

Rapid testing measures are critical for identifying symptomatic and asymptomatic carriers, guiding treatment and quarantine recommendations, and tracking viral spread. Rapid testing for SARS-CoV-2 has been predicted to have the greatest impact on transmission mitigation when deployed early in the onset of infections in a community [3–5]. Access to tests with quick time-to-result are critical for quelling the pandemic. Limited access to testing and long turnaround time have been reported to be contributing factors to poor pandemic control in some countries [6, 7]. Delays in diagnosis lead to corresponding delays in treatment decision, community spread and render contact tracing ineffective. Modeling suggests that providing decentralized, point-of-care testing with rapid feedback would have significantly greater impact on transmission vs. improving the absolute limit of detection of the assay [8]. Such models incorporate the observation that infectious SARS-CoV2 viral particles have not been recovered in culture below approximately 100 genomic copies/μL [8].

The pandemic has created an unprecedented global demand for RT-PCR testing. Direct antigen-based testing for SARS-CoV-2 may be a cost-effective alternative to RT-PCR anytime immediate results are advantageous, resources are constrained, or RT-PCR is not accessible. This is particularly important in low-to-middle income countries with severely limited access to RT-PCR. Testing rates in high-income settings are roughly ten times higher than those in low- and middle-income countries (LMICs) [9, 10]. Antigen tests to detect the presence of viral proteins can be performed directly on biological samples, such as tissue swabbed from the anterior nasal cavity, oropharynx, or even directly from saliva. Antigen tests are already used to detect influenza, strep throat, and other infectious diseases. LFA antigen tests can be rapidly produced in large volumes, i.e. billions of units/year. These tests are relatively inexpensive, require limited user training and are easy to administer; results are delivered in minutes. Importantly, unlike serological tests, LFA antigen tests directly detect pathogens and thus can be used to detect active infections. Although several SARS-CoV-2 direct antigen LFAs have been developed and commercialized, these commercial assays rely on proprietary designs, and are not available to generic LFA manufacturers in LMIC settings. The gap in testing rates between high-income countries and LMICs indicates that well-validated, cost-effective and sufficiently distributed tests are still lacking. Accordingly, access to tests in LMICs has been constrained by both cost and availability. The World Health Organization (WHO) highlights the need for local suppliers and technical support [11]–by providing an open-access architecture we aim to facilitate the manufacture of SARS-CoV-2 LFAs in places where they’re most needed. By selecting commercially sourced reagents and materials, we remove the barrier for novel reagent generation and validation.

In this paper we describe an open-access LFA for the detection of SARS-CoV-2 nucleocapsid protein, that can be mass-produced using commercially-available antibodies and materials. We combined the results of our previously reported high-throughput robotic antibody screening efforts [12] with our experience building a half-strip LFA for SARS-CoV-2 [13] in order to develop a complete LFA that utilizes anterior nares swabs. We report our limit of detection (LoD) against irradiated virus in simulated respiratory secretion (SRS).

## Materials and methods

### Six percent casein preparation

Casein for blocking latex beads was prepared by first mixing 6.183 g of boric acid (Sigma, St Louis, MO, B0394) and 800 mL of Ultrapure water (Invitrogen, Carlsbad, CA 10977–015), with stirring. The pH was adjusted to 8.5 using NaOH. Next, 100 g of casein sodium salt powder (Sigma, St Louis, MO, C8654) was added to the solution with continuous stirring. Using a heat plate, the temperature of the solution was brought to 40°C and held overnight. The following day, the solution was diluted to 1 L using ultrapure water and filtered through a Nalgene 0.2 μm filter device (VWR, Radnor, PA 89176–982). If the filter clogged, the solution was transferred to a fresh unit until the entire solution was filtered. The concentration of casein in the filtered solution was determined by measuring the residual mass of a 1mL aliquot after drying. The solution was diluted in 100 mM borate to bring the final stock concentration to 6% w/v and aliquoted and frozen at -20°C. This casein is used for the latex bead conjugation and nitrocellulose blocking protocols.

### One-day aged, 1% casein preparation

In a clean beaker, 0.82 mL of 50% NaOH (Sigma, St Louis, MO,415413) was added to 400 mL of Ultrapure H20 with stirring. Next, 5 g of casein (Sigma, St Louis, MO, C7078) was added to the solution and allowed to dissolve for 30 minutes with stirring. After the casein was fully dissolved, 1.2 g of boric acid (Sigma, St Louis, MO, B0394) and 2.2 g of sodium tetraborate decahydrate (Sigma, St Louis, MO, S9640) were added to the solution, with continuous stirring. The pH was adjusted to 8.5 using HCl. Ultrapure water was added to bring the volume to 500 mL. The solution was filtered using a Nalgene 0.2 μm filter unit and placed in a 40°C oven for 24 hours. The solution was aliquoted and frozen at -20°C until use. This casein is used for the conjugate-pad block and the conjugate diluent.

### Latex bead conjugation

For the test line conjugate, 400 nm carboxylic blue latex beads (Magsphere, Pasadena CA, USA, CAB400NM) were conjugated to Sino Biological 40143-R004 rabbit monoclonal antibody (Sino Biological, Beijing, China) at a weight:weight ratio of 30:1 (beads:antibody) using EDC/NHS coupling. For the control line conjugate, 400 nm carboxylic blue latex beads were conjugated to Chicken IgY (Jackson ImmunoResearch, West Grove PA, ChromPure 003-000-003), at a weight:weight ratio of 10:1 (beads:antibody) using EDC/NHS coupling.

Stock latex particles were vortexed and sonicated prior to use. The particles were then washed and resuspended in 0.1 M MES, pH 6.1 (Teknova, Hollister, CA, M225). The beads were covalently coupled to the antibodies in a two-step process. First, the beads are activated using freshly prepared EDC and NHS in MES. After 30 minutes, the beads were washed and resuspended in PBS pH 7.2. Antibodies were added at the ratios described above, and the activated beads were mixed with the antibodies for 3 hours with end-over-end mixing.

The conjugates were then quenched for thirty minutes using ethanolamine, again on the orbital shaker. Next, 6% casein was added to the mixture to a final concentration of 1% casein. The conjugates were blocked overnight at room temperature on the orbital shaker. The following day, conjugates were washed and stored in 50 mM borate with 1% casein (from the 6% latex block stock). Concentrations for the completed conjugates were determined by measuring the absorbance at 560 nm for red and 660 nm for blue. Final stocks were stored at 4°C until use.

### Antibody biotinylation

Sino Biologicals 40143-MM08 was first buffer exchanged into PBS to remove any interfering substances using Amicon filters (Sigma, St Louis, MO, 50 kDa MWCO, UFC5050). Specifically, the antibody was concentrated 20-fold and brought back to the original volume with PBS. This was done three times to remove sodium azide. The antibody was biotinylated at 1.5 mg/mL with 50 molar excess NHS-dPEG_12_-biotin (10198, Quanta Biodesign, Plain City, OH, USA). Excess biotin was removed using Amicon filters again, this time with five total concentration and resuspension cycles. The concentration of the biotinylated antibody was determined using the A280. The final biotinylation molar ratio was determined to be 20 moles of biotin per mole of antibody using the QuantTag Biotin Quantification Kit (Vector Laboratories, UK, BDK-2000).

### Nitrocellulose striping

The nitrocellulose, 25 mm CN95 (Sartorius), was striped with a test line at 8 mm from the edge of (upstream from the flow direction) and 13 mm from the upstream edge of nitrocellulose. The test line was striped at 1 μL/cm with 1 mg/mL polystreptavidin (Biotez, Berlin, DE Cat #10 120 050), and the control line was striped at 1 μL/cm 0.5 mg/mL goat anti-Chicken IgY (Jackson ImmunoResearch, West Grove, PA, 703-005-155),. Both lines were striped using a Biodot Frontline dispenser (Biodot, Irvine, CA, XYZ3060),. Strips were dried for one hour at 25°C and moved to an argon-purged desiccator overnight.

### Nitrocellulose blocking

Nitrocellulose strips were blocked in a solution containing 0.3% casein (from 6% stock), 5 mM borate (ThermoFisher, Wattham, MA, 28341), 2% w/v beta lactose (Sigma, St Louis, MO, L3750) and 2% w/v sucrose (Sigma, St Louis, MO, S7903).

To block, striped nitrocellulose was placed with the long edge submerged in the blocking solution. The solution was allowed to wick until the nitrocellulose was fully wet, at which point it was submerged fully in the blocking solution. The nitrocellulose remained in the blocking solution for 15 minutes, with continuous rocking. Finally, the nitrocellulose was removed from the blocking solution and placed in a 25°C forced-air oven for 30 minutes or until dry. It was then immediately moved to an argon-purged desiccator for storage.

### Conjugate pad blocking

A chopped glass fiber conjugate pad cut to 29 mm width (Lydall Performance Materials, Rochester, NH, Lypore 9818) and blocked with a solution of 0.075% one-day aged casein (from 1% stock), 5 mM borate (ThermoFisher, Wattham, MA, 28341), 0.05% Tween-20 (Seracare, Milford, MA 5460–0019) and 0.2% w/v sucrose (Sigma, St Louis, MO, S7903). The entire conjugate pad was submerged in the blocking solution for 15 minutes with continuous rocking. The nitrocellulose was removed and dried in a 40°C forced-air oven for 60 minutes.

### Sample pad blocking

The sample pad is a chopped glass fiber pad cut to 14 mm width (Ahlstrom-Munksjö, Helsinki, Finland, product 8964). The pad is blocked in 0.05% one-day aged casein (from 1% stock), 5 mM borate, 0.2% w/v sucrose, 0.05% w/v PVP-40 (Sigma, St Louis, MO, PVP-40) and 1 mg/mL HBR-1 (Scantibodies, Santee, CA). The sample pad was submerged completely in blocking solution with continuous rocking for 15 minutes and then dried at 25°C in a forced-air oven.

### Conjugate spraying and biotinylated antibody striping

The test-line and conjugate-line latex beads were sonicated and diluted in 1% one-day aged casein with 10% sucrose and 2% trehalose to a final concentration of 0.065% test-line beads and 0.015% control-line beads. The bead solution was sprayed in three discreet locations on the conjugate pad, 10, 15 and 20 mm from the upstream edge of the nitrocellulose. The conjugate was sprayed at 4 μL/cm using the Biodot Airjet (Biodot, Irvine, CA, ZX1010). Biotinylated 40143-MM08 was similarly diluted in 1-day aged casein with 10% sucrose and 2% trehalose to a final concentration of 75 μg/mL. It was striped at a rate of 4 μL/cm using the Biodot Frontline, 5mm from the upstream edge of the conjugate pad.

### Conjugate pad lyophilization

The conjugate pad was then immediately transferred to a lyophilizer (Advantage Pro, SP Scientific, Stone Ridge, NY) with shelves pre-cooled to -40°C. As soon as all the conjugate pads were transferred to the lyophilizer, the lyophilization cycle was started. The cycle began with 15 minutes of freezing at -35°C with no vacuum. Next, with the shelf temperature held at -35°C, vacuum was applied until the pressure reached 100 mTorr. After one hour, the temperature was increased to 20°C over the course of 55 minutes, while maintaining 100 mTorr pressure. Finally, the vacuum was reduced to 1000 mTorr until the pads were removed and placed directly in a desiccant cabinet.

### LFA assembly

**Fig 1:**
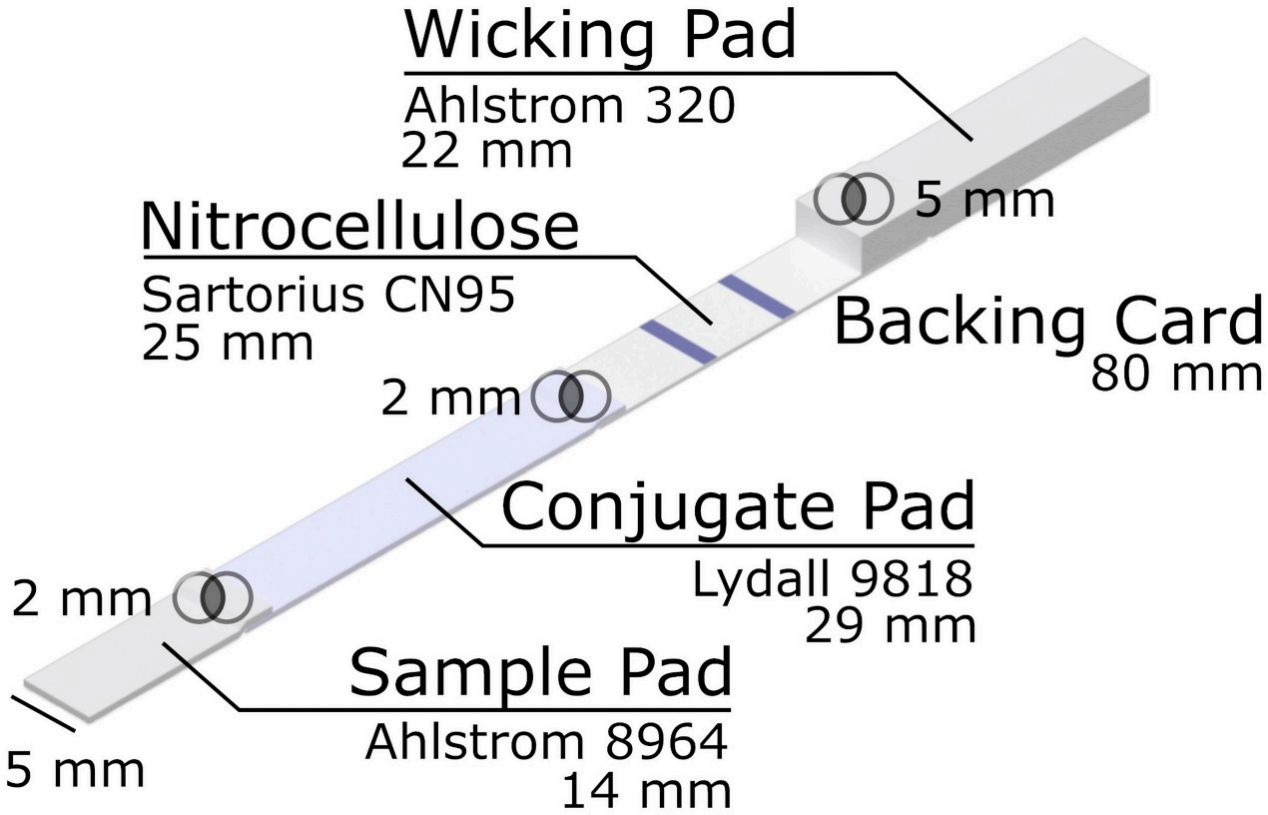
An LFA was constructed using 14 mm of a sample pad (Ahlstrom 8964), 29 mm of a conjugate pad (Lydall 9818), 25 mm of a nitrocellulose analytical membrane (CN95), and 22 mm of wicking pad (Ahlstrom 320). Intersecting circles indicate the overlap between two materials. The test line and control line are striped at 8 and 14 mm from the downstream end of the nitrocellulose membrane.

Blocked nitrocellulose was placed on an .002" thick 80 mm backing card (Lohmann). A 22 mm Ahlstrom 320 wicking pad (Ahlstrom-Munksjo Oyj, Finland) was placed on top of the downstream edge of the nitrocellulose with a 5 mm overlap between the two materials. The sprayed and blocked conjugate pad was placed on top of the upstream edge of the nitrocellulose with a 2 mm overlap. Finally, the sample pad is placed on top of the upstream edge of the conjugate pad with a 2 mM overlap. Strips were cut to a 5 mm width using a Kinematic Matrix guillotine cutter (Kinematic Automation, Inc., Twain Harte, CA, USA). A diagram of an assembled strip is shown in Fig 1. Strips were then placed in individual injection molded cassettes and stored in desiccator until use.

### Elution buffer

The elution buffer consists of 1X phosphate buffer saline (PBS) (ThermoFisher, Wattham, MA, 10010023) with 2% w/w Igepal CA-630 (Sigma, St Louis, MO, I8896). Each LFA is paired with 500 μL elution volume in a free-standing tube (VWR, Radnor, PA 16466–050).

### Swab selection

Three different swab types were evaluated for their ability to effectively release nucleocapsid protein into the extraction buffer within one minute. We selected a foam tip swab (Puritan Medical Products, Guilford, Maine, 25–1506 1PF BT), a flocked tip swab (Puritan Medical Products, Guilford, ME, 25-3806-U BT), and a spun polyester swab (SteriPack^™^, Lakeland, FL, 60567RevA).

The ability of the swab to release virus into the extraction buffer was tested by pipetting simulated respiratory secretion (SRS) spiked with gamma irradiated SARS-CoV-2 (BEI Resources, Manassas, VA, NR-52287, lot 70033322) directly onto each swab. Simulated respiratory secretion (SRS) was made based on the protocol described by Bose et al. [14]. Table 1 lists the specific concentrations and components used in our formulation. To test the relative release efficiency of each swab type, 20 μL of SRS with or without 2.5 x 10^7^ copies/mL (5 x 10^5^ copies/swab) irradiated virus was pipetted onto each swab. The swab was then placed into a 0.5 mL tube (VWR, Radnor, PA 16466–050) containing 500 μL of elution buffer, twirled three times, and incubated for one minute. Next, the swab was removed. Each swab type was tested in triplicate with both SRS alone and SRS with irradiated virus.

**Table 1:** Simulated respiratory secretion components.

Component	Final Concentration	Vendor and Product number
Na^+^	181 mM	Sigma S5886
K^+^	10 mM	Sigma P4504
Ca^++^	5 mM	Acros Organics 10043-52-4
Epithelial Cells	100,000 cells/mL	ATCC, CCL-185
Human Serum Albumin	0.64 mg/mL	Sigma A1653
IgG	706 μg/mL	Sigma 56834
IgM	86 μg/mL	Millipore AG722
Mucin	2.61 mg/mL	Sigma M3895

The eluted nucleocapsid protein concentration was determined using a sandwich immunoassay on the Meso Scale Discovery (MSD) platform (Meso Scale Diagnostics, Rockville, Maryland Meso QuickPlex SQ120). The immunoassay protocol is provided in supplemental information. **LOD Testing Protocol**.

The visual LOD was determined for both the assay described here [denoted open access LFA (OA-LFA)] and the Abbott^®^ BinaxNOW^™^ Covid-19 Ag Test. Serial dilutions of SARS-CoV-2 gamma irradiated virus (BEI NR-52287, lot 70033322) were made ibn SRS. Each dilution was tested in triplicate. Serial dilution of SARS-CoV-2 gamma irradiated virus (BEI Resources, Manassas, VA, NR-52287, lot 70033322) were made in SRS. Each dilution was tested in triplicate.

Next, 20 μL of diluted virus was pipetted onto a foam tip swab. For the OA-LFA, the swab was then placed in the elution buffer, twirled three times, and allowed to sit for one minute before being removed and discarded. 150 μL of the elution buffer was transferred to the sample port of the LFA using a disposable exact volume transfer pipette (ThermoFisher, Waltham, MA, 28341). After 30 minutes, quantitative test line intensities were measured using a commercial LFA reader (Axxin, Victoria, Australia AX-2X-s) and visually read. For the BinaxNOW^™^ test, the swab was processed per the test instructions. Briefly, six drops of extraction buffer were placed in the top hole of the swab well. Next, the swab was inserted into the bottom hole of the swab well and twirled three times. The adhesive liner was peeled off and the test was folded in half. The test was read after 15 minutes. Both tests were read visually by two independent readers. Each concentration was run in triplicate. Sample order was randomized, and readers were blinded to irradiated virus concentration and quantitative test line intensity values.

Initially, the lowest positive concentration tested was 0.51 TCID50/mL (3.13 x 10^3^ copies/mL). Because this concentration was consistently detected visually with the BinaxNOW^™^ test, subsequently 0.26 TCID50/mL (1.56 x 10^3^ copies/mL) was tested as well. In order to continue to blind the readers to the concentration, these three additional samples were randomized with three negative (0 copies/mL) samples.

### Limit of detection determination

The visual LOD was selected as the lowest concentration that both visual readers called positive for all three technical replicates. For further validation, we plotted the logarithmic value of the Axxin reader peak intensity versus the irradiated virus per swab. A cut-off value according to Eq 1 was utilized [15]. The visual LOD was utilized as the low concentration.


$$\mu_{neg}=1.65\sigma_{neg}+1.65\sigma_{low\;concentration}$$ (1)

For both test types, the LOD was confirmed by running an additional seven tests with SRS spiked at the LOD concentration randomized with SRS with no virus, for a total of ten replicates for both the LOD concentration and negative controls.

### Assessment of effect of extraction volume

To evaluate the hypothesis that extraction volume may affect signal intensity, we compared the performance of our LFA utilizing 500 μL vs 275 μL extraction buffer. For both elution volumes, 150 μL of eluent was transferred to the LFA using a standard variable volume pipette. For each elution volume, we ran the LFAs in triplicate with SRS and SRS spiked at with virus corresponding to the LOD concentration.

## Results

**Fig 2:**
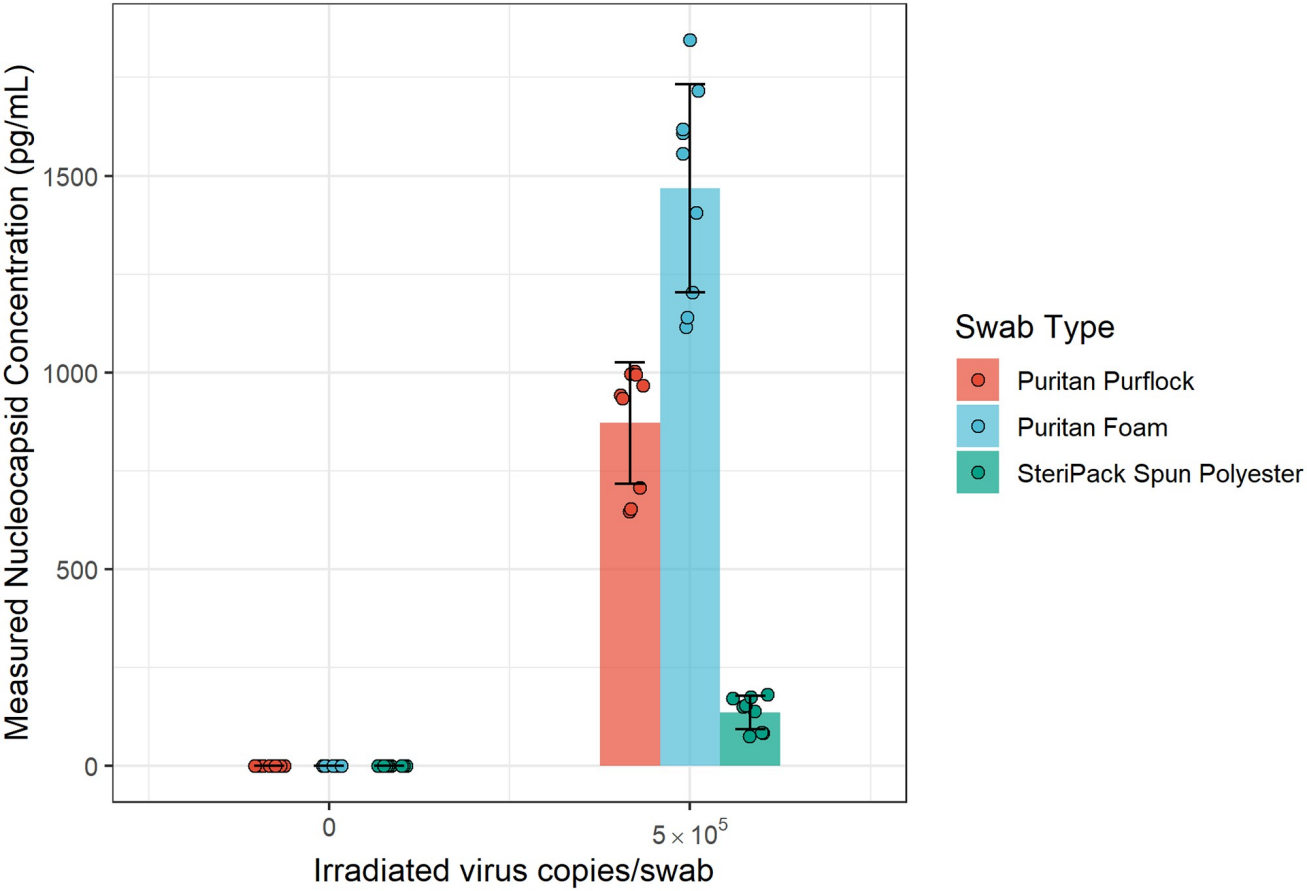
Measurable SARS-CoV-2 nucleocapsid released from three different swab types. SRS with or without SARS-CoV-2 was pipetted onto each swab before being placed in elution buffer. Eluted nucleocapsid protein was measured using the Meso Scale Discovery platform.

The concentration of measurable SARS-CoV-2 nucleocapsid protein eluted from each swab type is shown in Fig 2. Foam swabs showed the greatest concentration of eluted virus, followed by flocked swabs. There was a dramatic decrease in released nucleocapsid for the spun polyester swabs. Foam swabs were utilized for the LOD testing.

**Fig 3:**
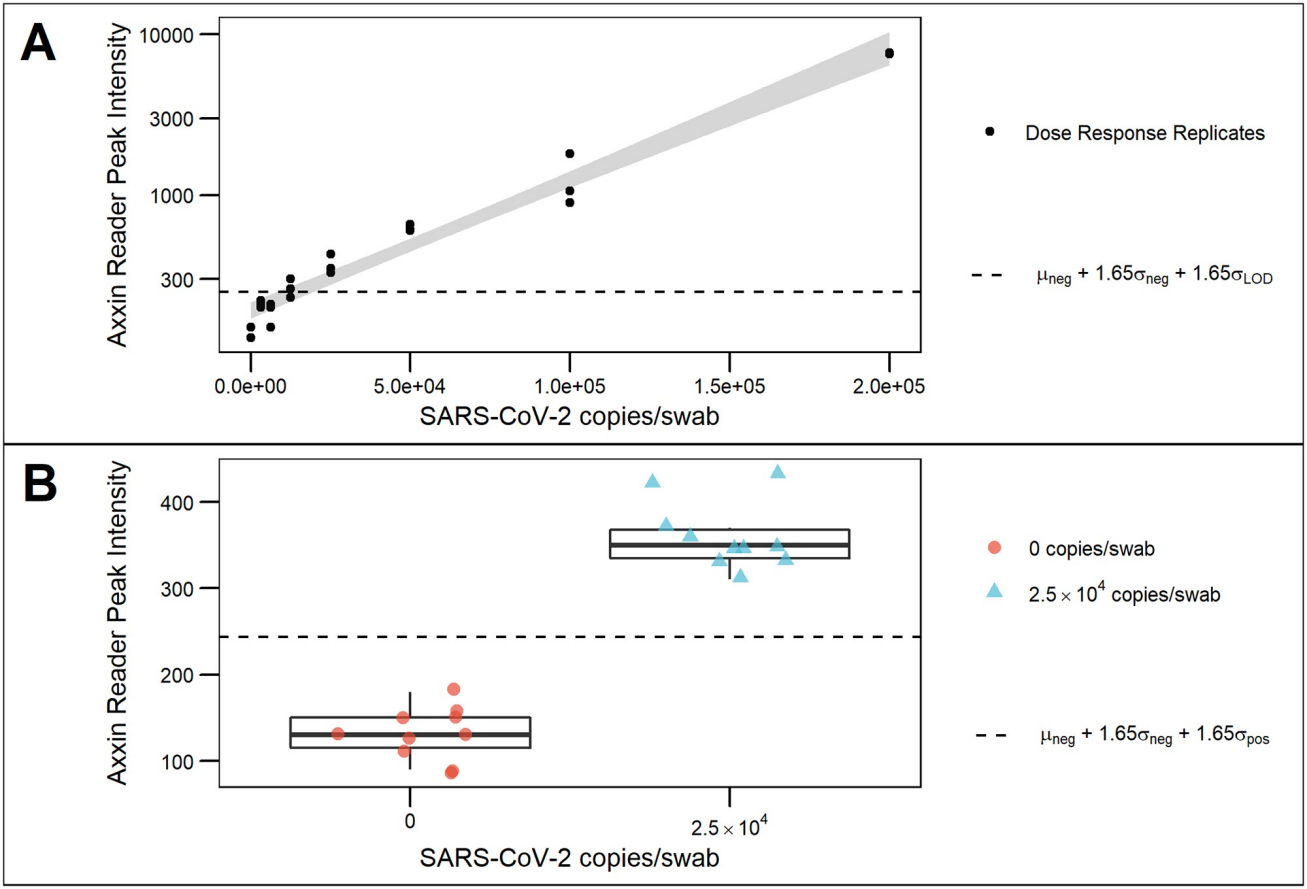
Analytical performance of OA-LFA. **(A)** Dose-response curve of gamma-irradiated virus spiked into SRS. Concentrations refer to the total amount of genomic material applied to the swab. **(B)** Validation of LOD using ten negative replicates and ten replicates spiked with SARS-CoV-2 at the LOD concentration.

The visual read results of the LOD experiment are provided in Table 2. The OA-LFA reported herein has an LOD of 4 TCID_50_/swab, equivalent to 2.5 x 10^4^ copies/swab. The dose-response curve, generated using the peak test line intensity score from the Axxin reader, is shown in Fig 3A. This curve does not include the lowest tested concentration (0.26 TCID50/mL) (1.56 x 10^3^ copies/mL), as this was tested separately. Further details are available in the supplemental materials. Direct comparison with BinaxNOW^™^ showed that BinaxNOW^™^ has a lower LOD (0.5 TCID_50_/swab). The additional seven replicates at the LOD concentration randomized with SRS alone confirmed the LOD of both tests, with readers identifying the negative and positive strips with 100% accuracy for both BinaxNOW and the OA-LFA. The peak test-line intensity for the 10 negatives and 10 replicates at the LOD concentration are shown in Fig 3B. Although ultimately designed to be a visually read test, the Axxin value reader values show that all test-line intensity for the LOD concentration are above the limit-of-detection signal (denoted by the dotted horizontal line).

**Table 2:** Visual LOD determination. The visual LOD for the OA-LFA test is 4 TCID_50_/swab and the BinaxNOW^™^ LOD is 0.5 TCID_50_/swab.

Virus concentration	Virus Concentration	OA-LFA Visual Read Fraction Positive	BinaxNOW^™^ Visual Read Fraction Positive
(Viral Copies/swab)	(TCID_50_/swab)		
0	0	0/20	0/20
1.56×10^3^	0.26	0/6	5/6
**3.13×10** ^**3**^	**0.51**	**0/6**	**20/20**
6.25×10^3^	1.03	0/6	6/6
1.25×10^4^	2.06	1/6	6/6
**2.50×10** ^**4**^	**4.12**	**20/20**	**6/6**
5.00×10^3^	8.24	6/6	6/6
1.00×10^3^	16.47	6/6	6/6
2.00×10^3^	32.94	6/6	6/6

### Effect of elution volume

**Fig 4:**
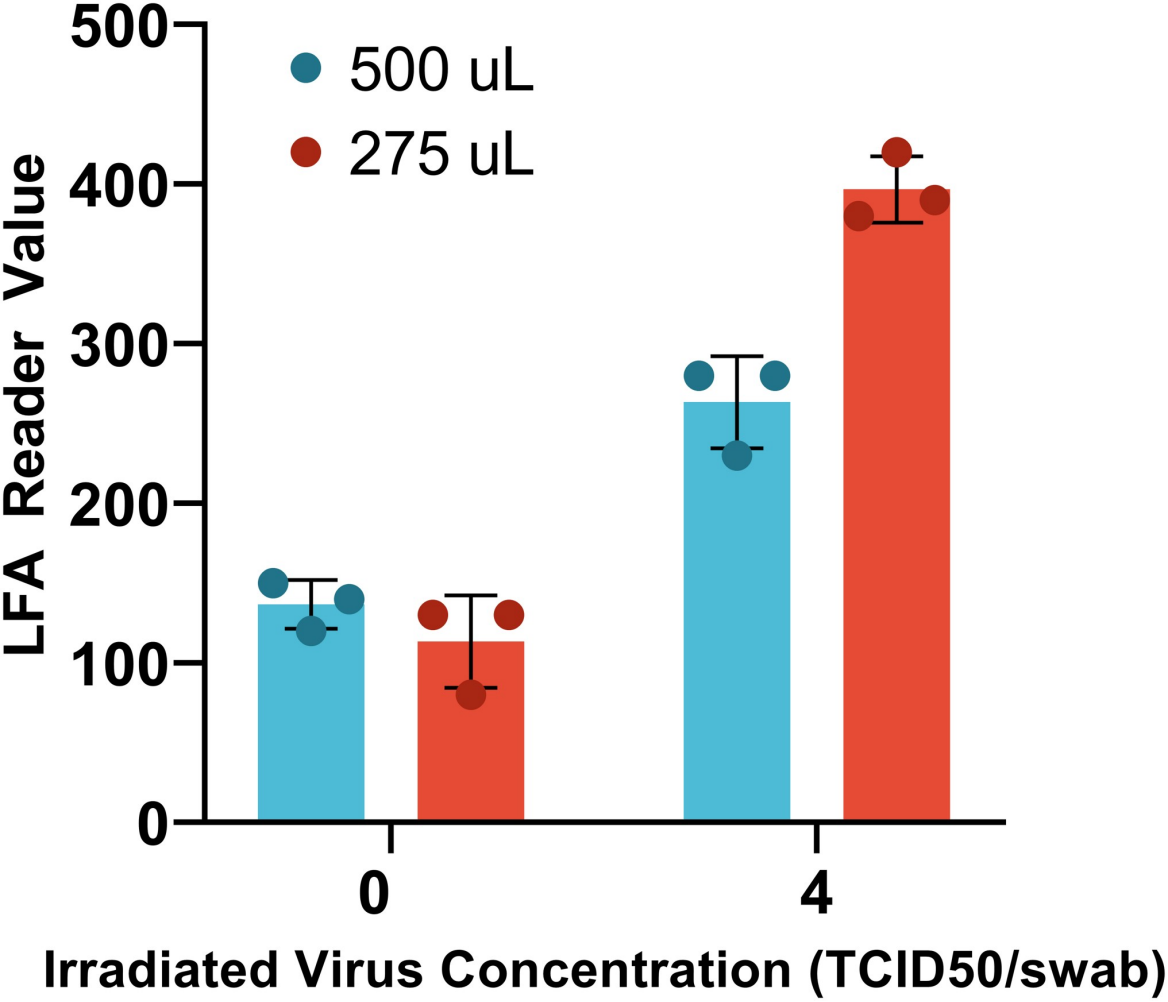
Comparison of diluent volume. Swab was placed into a tube containing either 275 or 500 μL of sample diluent and run using the conditions described previously. Each condition was tested with a negative control and irradiated virus diluted in simulated respiratory secretion, run with an n = 3. Reducing the dilution factor increases the signal near our LOD, suggesting an improvement in overall performance.

One notable feature of the Abbott BinaxNOW^™^ test is the use of a minimal swab extraction buffer volume, 6 drops or approximately 150 μL, all of which is applied to the LFA. We hypothesize that their superior analytical sensitivity is due, in part, to the significantly lower extraction volume. To test this hypothesis, a lower elution volume was evaluated. As shown in Fig 4, reducing the extraction volume to 275 μL increased the test-line intensity by 60% at the LOD concentration (4 TCID_50_/swab), without increasing the background signal.

## Discussion

Here we describe a detailed SARS-COV2 N antigen LFA with an LOD that meets the acceptable limits of the WHO target product profile (TPP) [16] Specifically, the WHO TPP states that the acceptable analytical LOD is 10^6^ copies/mL. Assuming a typical extraction volume of 3 mL, this corresponds to an LOD of 3.3×10^5^ copies/swab, roughly an order of magnitude higher than the LOD of the OA-LFA reported here. While the analytical LOD is inferior to that of BinaxNOW^™^ Covid Ag test, our LOD is similar to other tests granted FDA EUA status. For example, the BD Veritor SARS-CoV-2 reports their LOD to be 7 TCID_50_/swab using gamma irradiated virus [17]. Furthermore, the OA-LFA meets the WHO TPP [16] acceptable targets for time to result, number of user steps and target use setting. The test meets the WHO desirable criteria for end user profile because it can readily be used by trained staff in community level health centers; the test is simple to use [18] and operates similarly to ubiquitous malaria rapid tests.

We report our LOD here in both SARS-CoV-2 viral copies/swab and TCID_50_/swab. FDA EUA submissions for antigen tests have reported sensitivity in terms of TCID_50_; the TCID_50_ is the concentration at which 50% of cells are infected when a test tube or well plate upon which cells have been cultured is inoculated with a diluted solution of viral fluid. However, the ratio between viral copy and TCID_50_ varies significantly between lots of gamma-irradiated virus and care must be taken when comparing results from different lots and preparations of viruses. Additionally, the clinical performance of the open-access LFA described herein has been reported separately; the LFA demonstrated 69% sensitivity and 97% specificity relative to nasopharyngeal (NP) PCR and 83% sensitivity and 97% specificity relative to anterior nares (AN) PCR [18]. The superior analytical sensitivity of the BinaxNOW^™^ Covid Ag test translated to improved clinical sensitivity; however, the OA-LFA performed equivalently to the Quidel Sofia^®^ Covid test. Relative to AN PCR, the OA-LFA met the WHO TPP acceptable sensitivity target. Crucially, the LFA described here can be considered an open-access design for an antigen-detecting point of care test for SARS-CoV-2, as it is designed entirely from commercially sourced materials. If antibody supplies for the antibodies chosen herein are limited or excessively costly, our previously reported extended antibody screening data demonstrated excellent alternatives and can be utilized to evaluate alternate choices [12]. The information we present can be used by any organization to develop an LFA for both validation and towards large-scale production.

Our swab selection only looked at a swab’s ability to effectively release absorbed nucleocapsid protein into the elution buffer. However, the other component in a swab’s efficacy is its ability to collect virus in the *anterior nares*. It is possible that flocked swabs, designed to collect more specimen, would outperform foam swabs in practice. However, the poor extraction efficiency exhibited by the SteriPack^™^ spun polyester make them a poor choice for antigen-based SARS-CoV-2 assays. For the purposes of benchmarking our test, the foam swab was chosen due to its highly efficient elution. Additionally, the BinaxNOW^™^ test utilized the same foam swab, removing this variable from the comparison in analytical performance.

This test was designed with a buffer compatible with anterior nares swabs. Future work should confirm its suitability in other sample matrices, including saliva. This first-generation product can likely benefit from further optimization and improved performance through higher sensitivity nanoparticles for detection, improved extraction buffer design for optimal exposure of the target epitopes and/or reduced dilution volume. While the use of reduced extraction volume is a promising approach, this would necessitate a different transfer modality than the exact volume transfer pipet used here. Various other assays have instead utilized an extraction buffer dropper bottle, so this is a relatively easy adjustment.

Interestingly, we found that the LOD for the Abbott BinaxNOW^™^ test was 0.5 TCID_50_/swab, substantially lower than the LOD reported in the BinaxNOW^™^ EUA submission (22.5 TCID_50_/swab). We believe this discrepancy may be due to heat-inactivation of the viral standard, as reported in the BinaxNOW^™^ EUA submission, in comparison to the gamma-irradiated viral standard used here. Internal ELISA testing has shown that gamma-irradiated virus produces significantly greater signal that heat inactivated virus, which we speculate may be due to heat-mediated aggregation. In comparing analytical sensitivity of available antigen-based tests, it’s imperative to ensure the same reference standards are utilized.

There are several limitations of this study. First, a cost analysis is not provided. Due to the fact that our organization is not a large-scale LFA manufacturer, we are unable to accurately assess costs at scale. Secondly, the accessibility of commercially available antibodies can vary by country. We believe that the architecture described in this manuscript, coupled with the antibody screening previously reported [12], will provide an array of options for potential assay manufacturers. Additionally, although a direct analytical comparison to the BinaxNOW^™^ test is presented, we were not able to provide direct comparison with other FDA EUA Covid-19 antigen tests. Due to the variability in inactivated virus performance, it is difficult to compare analytical sensitivity between tests not assessed directly against one another. To help mitigate this, we have provided our testing procedure and materials in sufficient details for other researchers to replicate. Finally, stability was assessed utilizing accelerated stability testing at 40°C through 31 days. Although the LFAs were stable through this time period (results show in S5 Fig), no further stability testing was performed. Ultimately, stability testing will need to be performed by final assay manufacturers as ambient temperature and humidity during production and packaging of LFAs can have an effect on test stability.

## Conclusion

In this paper we present an open-access LFA designed for the detection of the nucleocapsid protein of SARS-CoV-2. Importantly, it meets the stated analytical sensitivity requirements proposed by WHO [16]. We utilize only commercially sourced reagents and conventional protocols, to allow the straightforward duplication and modification of this LFA. The LOD of this assay meets the requirements stated by the WHO for use as a POC test. Further work, including validation of the assay in a clinical setting and its performance using different specimen types will be reported elsewhere.

## Supporting information

S1 File Supplemental methods and full datasets for results provided in the main text. (DOCX)

S1 Fig Concentration optimization of biotinylated antibody spotted onto the conjugate pad, tested using an n = 3. The steep drop-off after 18.5 mg/mL of biotinylated antibody suggests that at those lower concentrations the reaction is limited by this reagent. Therefore, a higher concentration of biotinylated antibody was selected for the final device.
(TIF)

S2 Fig Concentration optimization testing combinations of biotinylated antibody and latex conjugate. Conjugate pads were sprayed at different rates for the same concentration of latex conjugate, thereby changing the amount of conjugate deposited on each pad. Similar to S1 Fig, there is a steep drop off in signal as biotinylated antibody concentration is decreased. Changes in the speed of deposition for the latex conjugate, however, do not make as significant a difference.
(TIF)

S3 Fig Optimization of nitrocellulose striping speed: As it decreases the signal of the positive similarly. With regards to signal of the negative, the data sets can be grouped into two, with a drop off occurring between 0.8 μL/cm and 0.7 μ L/cm. In our final build, we used 1 μL/cm to maximize positive signal, and found alternate methods to reduce the signal in the negative, including nitrocellulose block and using a more hydrophobic conjugate pad (Lydall 9819).
(TIF)

S4 Fig Nitrocellulose blocking for improved membrane stability. The specifics of each nitrocellulose block can be seen in S1 Table in S1 File. These membranes were stored at 40°C in a sealed mylar bag containing desiccant. Reduced performance is seen in nitrocellulose block 2 and 4 at Day 0. By Day 21, Block 6 and the unblocked condition show the biggest drop off in signal (32.1 and 39.6% respectively). Blocks 5 and 7, both of which contain 2% sucrose and 2% beta lactose with differing amounts of casein and BSA.
(TIF)

S5 Fig Long term stability of final LFA build. Similar performance was seen when strips were tested at Day 0, Day 4, and Day 31 after being stored at 40°C.
(TIF)
