# Supplementary material for: A SARS-CoV-2 coronavirus nucleocapsid protein antigen-detecting lateral flow assay
Source: PLoS One. 2021 Nov 10;16(11):e0258819. doi: 10.1371/journal.pone.0258819 (PMC8580225; doi:10.1371/journal.pone.0258819)
Supplement: S1 File — (DOCX) [file pone.0258819.s001.docx]

S1 File:

**Title**: A SARS-CoV-2 Coronavirus Nucleocapsid Protein Antigen-Detecting Lateral Flow Assay

Authors

**Short Title:** Open-access Covid-19 rapid test

Benjamin D. Grant^1^*, Caitlin E. Anderson^1^, Luis F. Alonzo^1^, Spencer H. Garing^1^, John R. Williford^2,#a^, Ted. A. Baughman^1^, Rafael Rivera^1^, Veronika A. Glukhova^1^, David S. Boyle^3^, Puneet K. Dewan^1^, Bernhard H. Weigl^1^, Kevin P. Nichols^1,#b^

1 - Global Health Labs, Bellevue, Washington, USA

2 - Intellectual Ventures Lab, Bellevue, Washington, USA

3 - PATH, Seattle, Washington, USA

^#a^ Current Address: Nortis Inc, Woodinville, Washington, USA

^#b^ Current Address: Amazon Dx, Seattle, Washington, USA

* Corresponding author: ben.grant@ghlabs.org

**Table of Contents:**

Supplemental Table S1. Components in nitrocellulose blocks for improved membrane stability testing

Supplemental Methods: Meso Scale Diagnostics Nucleocapsid Immunoassay

Supplemental Data: Limit-of-detection

Supplemental Table S2: Axxin reader scores for limit-of-detection curve

Supplemental Table S3: Axxin reader score for limit-of-detection validation

Supplemental Data: Swab Selection

Supplemental Table S4: MSD results for swab selection

Supplemental Data: Elution Volume

Supplemental Table S5: Impact of elution volume on LFA performance

**Supplemental** **Table S1.** Table describing the components in each nitrocellulose block used for membrane stability testing. See **Supplemental** **Fig S4**.

| **​** | **Borate​** | **Casein​** | **Sucrose​** | **Beta lactose​** | **SLS​** | **Tween 20​** | **BSA​** |
| --- | --- | --- | --- | --- | --- | --- | --- |
| Block 1​ | 0.05 mM​ | 0.05%​ | 2%​ | -​ | -​ | -​ | -​ |
| Block 2​ | 0.05 mM​ | 0.05%​ | 1%​ | -​ | -​ | -​ | -​ |
| Block 3​ | 0.05 mM​ | 0.05%​ | 2%​ | 2%​ | -​ | -​ | -​ |
| Block 4​ | 0.05 mM​ | 0.05%​ | 2%​ | 0.17%​ | -​ | -​ | -​ |
| Block 5​ | 0.05 mM​ | 3%​ | 2%​ | 2%​ | -​ | -​ | -​ |
| Block 6​ | 0.05 mM​ | 0.05%​ | 2%​ | 2%​ | 0.05%​ | 0.25%​ | -​ |
| Block 7​ | 0.05 mM​ | 0.05%​ | 2%​ | 2%​ | 0.05%​ | -​ | 1%​ |

**Supplemental Methods S1: Meso Scale Diagnostics SARS-CoV-2 Nucleocapsid Assay**

The sandwich assay utilizes two monoclonal antibodies, 40143-MM08 and 40143-MM05 (Sino Biologicals, Beijing, China.), that exhibit high specificity to SARS-CoV and SARS-CoV-2 nucleocapsid proteins. Antibody 40143-MM08 is biotinylated with a long-spacer arm biotin (ThermoFisher, Waltham, MA, A35358). The antibody is incubated with 10:1 molar ratio biotin:antibody for two hours at RT. After two hours, excess biotin is removed utilizing Amicon 50 kDa molecular weight cutoff (MWCO) spin columns (Sigma, St. Louis, MO, UFC505096). Antibody 40143-MM05 is conjugated to a gold sulfo-tag (Meso Scale Diagnostics, Rockville, MD, R31AA-1) at a 20:1 gold sulfo-tag:antibody ratio. Excess tag is removed using an Amicon 50 kDa MWCO spin column. Final concentrations of both conjugated antibodies are determined using a bicinchoninic acid assay (ThermoFisher, Waltham, MA, 23225) using a Bovine Gamma Globulin standard (ThermoFisher 23212, Waltham, MA,).

The standard curve was created by spiking recombinant nucleocapsid protein (Acro Biosystems, Newark, DE NUN-C5227) into elution buffer. The top of the standard curve is 20 ng/mL. This is serial diluted in five-fold dilutions using elution buffer to a final concentration of 1.28 pg/mL for the lowest point on the standard curve. Elution buffer with no nucleocapsid protein serves as the negative.

Small spot streptavidin plates (Meso Scale Diagnostics, Rockville, MD, L45SA-1) are blocked with 150 µL/well of 5% blocker A (Meso Scale Diagnostics, Rockville, MD, R93BA) in 1x PBS. After adding blocker to each well, the plates are placed on a plate shaker for 1 minute at 700 rotations per minute (RPM). The plates are then taken off the shaker and incubated for 1 hour. Next, the plates are washed three times with 300 µL/well of wash buffer: 1X PBS with .05% TW-20 (Teknova, Hollister, CA P0197) using an automated plate washer (Biotek, Winooski, VT, 405 TS). Next, 25 µL/well of 0.5 µg/mL of biotinylated 40143-MM08 in 1X PBS with 1% BSA is added to each well. The plate is incubated on a plate shaker at 700 RPM for 1 hour. The plate is again washed three times with wash buffer. Next, 25 µL of each sample or standard is added per well. Each sample and standard are run in triplicate wells. Again, the plate is incubated for 1 hour on a plate shaker at 700 RPM. The plate is then washed three times with wash buffer. After washing, 25 µL of 0.31 µg/mL gold sulfo-tagged 40143-MM05 in 1% BSA in 1X PBS is added per well. The plate is incubated for 1 hour at 700 RPM on a plate shaker. The plate is then washed three times. Finally, 150 µL of MSD gold read buffer A (Meso Scale Diagnostics, Rockville, MD, R92TG) is added per well. The plate is read on the Meso Scale Quickplex SQ 120.

To determine the concentration of eluted nucleocapsid in the unknown samples, the standard curve is fit using a 5-parameter logistic fit with 1/(signal^1/2^) weighting. This fit was done using the DRC package in R. Based on this fit, concentrations for the unknowns can be determined based on their signal intensity.

**Supplemental Data: Limit-of-detection**

**Limit-of-detection experiment one**

Irradiated virus was diluted to a concentration of 1.00x10^7^ irradiated virus/mL in 400 µL of SRS. It was further serial diluted 1:2 down to the lowest concentration of 1.56x10^5^ copies/mL. Because 20 µL is pipetted per swab, this corresponds to a range of 3.13x10^3^ to 2.00x10^5^ copies/swab. A negative control (SRS alone) was included as the final concentration. Each concentration was run in triplicate. Each replicate of every concentration was assigned a random letter (A-X). Randomization was done independently for the BinaxNOW™ and OA-LFA tests. After running each sample, the LFAs were read visually by two independent researchers. Finally, an objective test-line intensity was obtained using an Axxin LFA reader. The resultant Axxin reader scores are shown below, in **supplemental** **table S2**. In the main text **Fig 3A**, the fit shown is a linear fit between the log of the Axxin Reader Signal and the concentration/swab. The fit has an intercept of 5.23 and a slope of 1.875x10^-5^. The Pearson’s correlation coefficient is 0.985.

**Supplemental Table S2: Axxin reader scores for limit-of-detection curve.**

| Concentration (copies/swab) | Axxin Reader Signal |
| --- | --- |
| 0 | 150 |
| 0 | 130 |
| 0 | 150 |
| 3125 | 220 |
| 3125 | 200 |
| 3125 | 210 |
| 6250 | 210 |
| 6250 | 150 |
| 6250 | 200 |
| 12500 | 300 |
| 12500 | 260 |
| 12500 | 230 |
| 25000 | 330 |
| 25000 | 350 |
| 25000 | 430 |
| 50000 | 660 |
| 50000 | 600 |
| 50000 | 610 |
| 100000 | 1810 |
| 100000 | 900 |
| 100000 | 1060 |
| 200000 | 7670 |
| 200000 | 7510 |
| 200000 | 7630 |

**Limit-of-detection experiment two**

The visual results revealed that all three replicates of the 3.13x10^3^ copies/swab condition were detectable by the BinaxNOW™ test. To ensure the true BinaxNOW™ LOD was not below this concentration, a subsequent experiment was run. In this experiment, three replicates of 1.56x10^3^ copies/swab and three replicates of SRS alone were randomized as described above and run on both the OA-LFA and BinaxNOW™ test.

**Limit-of-detection experiment three**

As shown in the main text **Table 1,** the lowest concentrations detected with 100% accuracy by both visual readers for the OA-LFA and BinaxNOW™ LFA were 2.50x10^4^ copies/swab and 3.13x10^3^ copies/swab, respectively. To validate these LODs, seven more replicates of each, randomized with 7 negative controls, were run as above. As shown in the main text, the visual read results confirmed the LOD determination. The OA-LFAs were again read with the Axxin reader. Results are shown below in supplemental table S3.

**Supplemental Table S3**: Axxin reader score for limit-of-detection validation

| Concentration (copies/swab) | Axxin Reader Signal | Experiment |
| --- | --- | --- |
| 0 | 150 | Limit-of-detection experiment 1 |
| 0 | 130 | Limit-of-detection experiment 1 |
| 0 | 150 | Limit-of-detection experiment 1 |
| 0 | 110 | Limit-of-detection experiment 3 |
| 0 | 90 | Limit-of-detection experiment 3 |
| 0 | 180 | Limit-of-detection experiment 3 |
| 0 | 160 | Limit-of-detection experiment 3 |
| 0 | 130 | Limit-of-detection experiment 3 |
| 0 | 130 | Limit-of-detection experiment 3 |
| 0 | 90 | Limit-of-detection experiment 3 |
| 25000 | 330 | Limit-of-detection experiment 1 |
| 25000 | 350 | Limit-of-detection experiment 1 |
| 25000 | 430 | Limit-of-detection experiment 1 |
| 25000 | 330 | Limit-of-detection experiment 3 |
| 25000 | 360 | Limit-of-detection experiment 3 |
| 25000 | 350 | Limit-of-detection experiment 3 |
| 25000 | 350 | Limit-of-detection experiment 3 |
| 25000 | 420 | Limit-of-detection experiment 3 |
| 25000 | 370 | Limit-of-detection experiment 3 |
| 25000 | 310 | Limit-of-detection experiment 3 |

**Supplemental Data: Swab Selection**

Swabs were compared as described in the main text. The MSD calculated concentrations are shown below in **supplemental table S4**.

**Supplemental Table S4.** Nucleocapsid concentration was measured using the MSD, testing with three different swab types, Puritan Foam, SteriPack Spun Polyester Swabs, and Puritan Flocked Swabs. A known concentration of nucleocapsid was added to each swab, at 500,000 copies/swab. Each condition was tested with an n=9.

| Concentration (copies/swab) | Measured Nucleocapsid Concentration (pg/mL) | Swab Type |
| --- | --- | --- |
| 500000.00 | 1845.556602 | Puritan Foam |
| 500000.00 | 1405.566102 | Puritan Foam |
| 500000.00 | 1115.379161 | Puritan Foam |
| 500000.00 | 1608.414679 | Puritan Foam |
| 500000.00 | 1555.965441 | Puritan Foam |
| 500000.00 | 1140.337557 | Puritan Foam |
| 500000.00 | 1716.669329 | Puritan Foam |
| 500000.00 | 1618.674055 | Puritan Foam |
| 500000.00 | 1203.27686 | Puritan Foam |
| 500000.00 | 151.2652897 | SteriPack Spun Polyester Swabs |
| 500000.00 | 182.4376201 | SteriPack Spun Polyester Swabs |
| 500000.00 | 84.17232916 | SteriPack Spun Polyester Swabs |
| 500000.00 | 139.5268272 | SteriPack Spun Polyester Swabs |
| 500000.00 | 171.6641889 | SteriPack Spun Polyester Swabs |
| 500000.00 | 75.65863322 | SteriPack Spun Polyester Swabs |
| 500000.00 | 154.5500285 | SteriPack Spun Polyester Swabs |
| 500000.00 | 175.8808348 | SteriPack Spun Polyester Swabs |
| 500000.00 | 86.06279514 | SteriPack Spun Polyester Swabs |
| 500000.00 | 707.6218195 | Puritan Flocked Swabs |
| 500000.00 | 647.4871037 | Puritan Flocked Swabs |
| 500000.00 | 653.4804895 | Puritan Flocked Swabs |
| 500000.00 | 942.3067126 | Puritan Flocked Swabs |
| 500000.00 | 934.5041094 | Puritan Flocked Swabs |
| 500000.00 | 1002.636089 | Puritan Flocked Swabs |
| 500000.00 | 995.9860151 | Puritan Flocked Swabs |
| 500000.00 | 967.0862717 | Puritan Flocked Swabs |
| 500000.00 | 994.380744 | Puritan Flocked Swabs |
| 0.00 | 0 | Puritan Foam |
| 0.00 | 0 | Puritan Foam |
| 0.00 | 0 | Puritan Foam |
| 0.00 | 0 | Puritan Foam |
| 0.00 | 0 | Puritan Foam |
| 0.00 | 0 | Puritan Foam |
| 0.00 | 0 | Puritan Foam |
| 0.00 | 0 | Puritan Foam |
| 0.00 | 0 | Puritan Foam |
| 0.00 | 0 | SteriPack Spun Polyester Swabs |
| 0.00 | 0 | SteriPack Spun Polyester Swabs |
| 0.00 | 0 | SteriPack Spun Polyester Swabs |
| 0.00 | 0 | SteriPack Spun Polyester Swabs |
| 0.00 | 0 | SteriPack Spun Polyester Swabs |
| 0.00 | 0 | SteriPack Spun Polyester Swabs |
| 0.00 | 0 | SteriPack Spun Polyester Swabs |
| 0.00 | 0 | SteriPack Spun Polyester Swabs |
| 0.00 | 0 | SteriPack Spun Polyester Swabs |
| 0.00 | 0 | Puritan Flocked Swabs |
| 0.00 | 0 | Puritan Flocked Swabs |
| 0.00 | 0 | Puritan Flocked Swabs |
| 0.00 | 0 | Puritan Flocked Swabs |
| 0.00 | 0 | Puritan Flocked Swabs |
| 0.00 | 0 | Puritan Flocked Swabs |
| 0.00 | 0 | Puritan Flocked Swabs |
| 0.00 | 0 | Puritan Flocked Swabs |
| 0.00 | 0 | Puritan Flocked Swabs |

**Supplemental Data: Elution Volume Comparison**

The effect of elution volume was evaluated as described in the main text. The resulting Axxin reader scores are shown in **supplemental table S5**.

**Supplemental Table S5.** Impact of elution volume on LFA performance. Table contains raw Axxin values for swab elution experiment. Irradiated virus was tested at 0 or 4 TCID50/swab. The volume in which the swab was eluted was varied between 500 and 275 µL. Each condition was tested with an n=3.

| TCID50/swab | 500 µL | | | 275 µL | | |
| --- | --- | --- | --- | --- | --- | --- |
| 0 | 150 | 140 | 120 | 130 | 80 | 130 |
| 4 | 280 | 230 | 280 | 420 | 380 | 390 |
