# Supplementary figures and images for: A SARS-CoV-2 coronavirus nucleocapsid protein antigen-detecting lateral flow assay
Source: PLoS One. 2021 Nov 10;16(11):e0258819. doi: 10.1371/journal.pone.0258819 (PMC8580225; doi:10.1371/journal.pone.0258819)

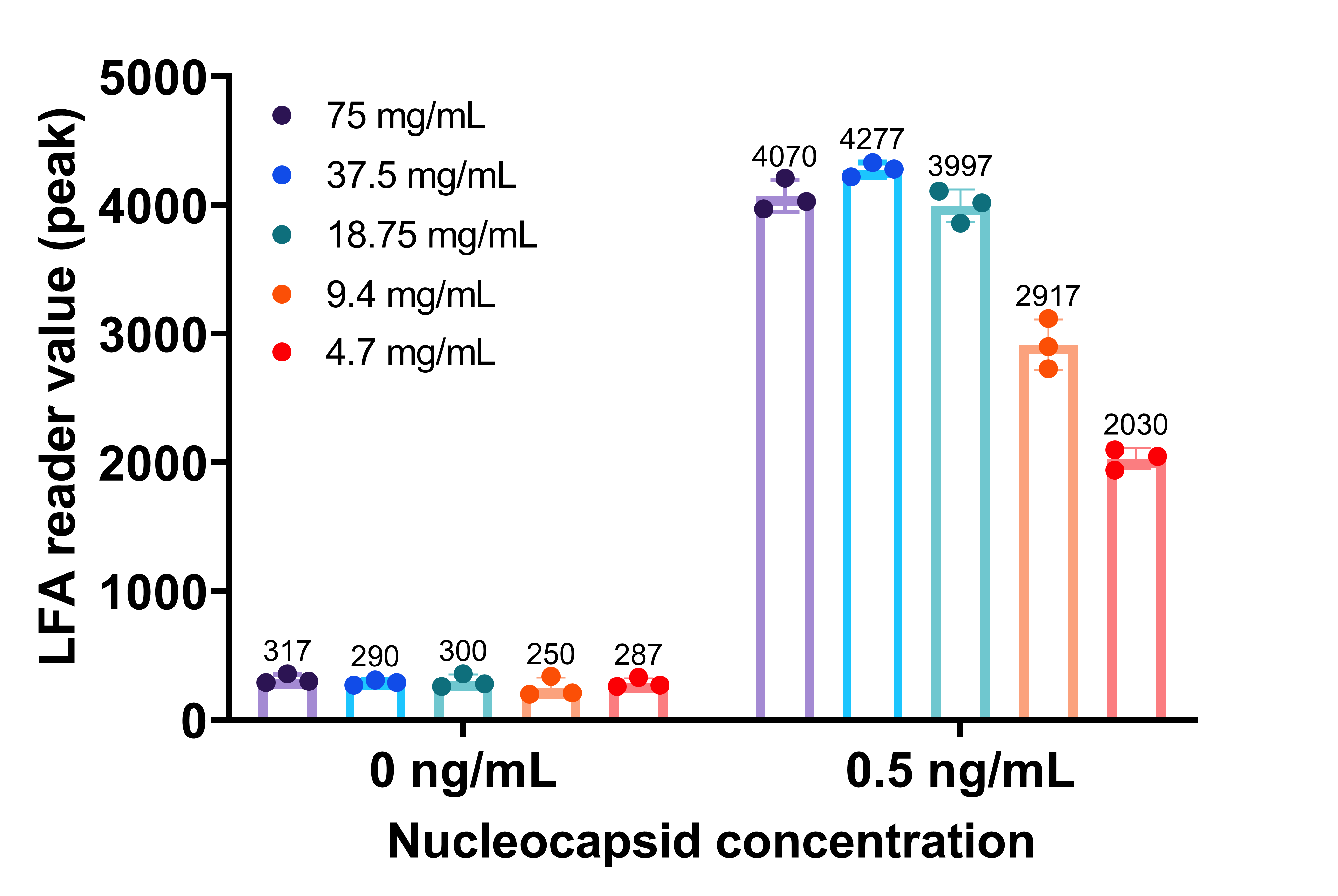

Supplement: S1 Fig — The steep drop-off after 18.5 mg/mL of biotinylated antibody suggests that at those lower concentrations the reaction is limited by this reagent. Therefore, a higher concentration of biotinylated antibody was selected for the final device. (TIF) [file pone.0258819.s002.tif]

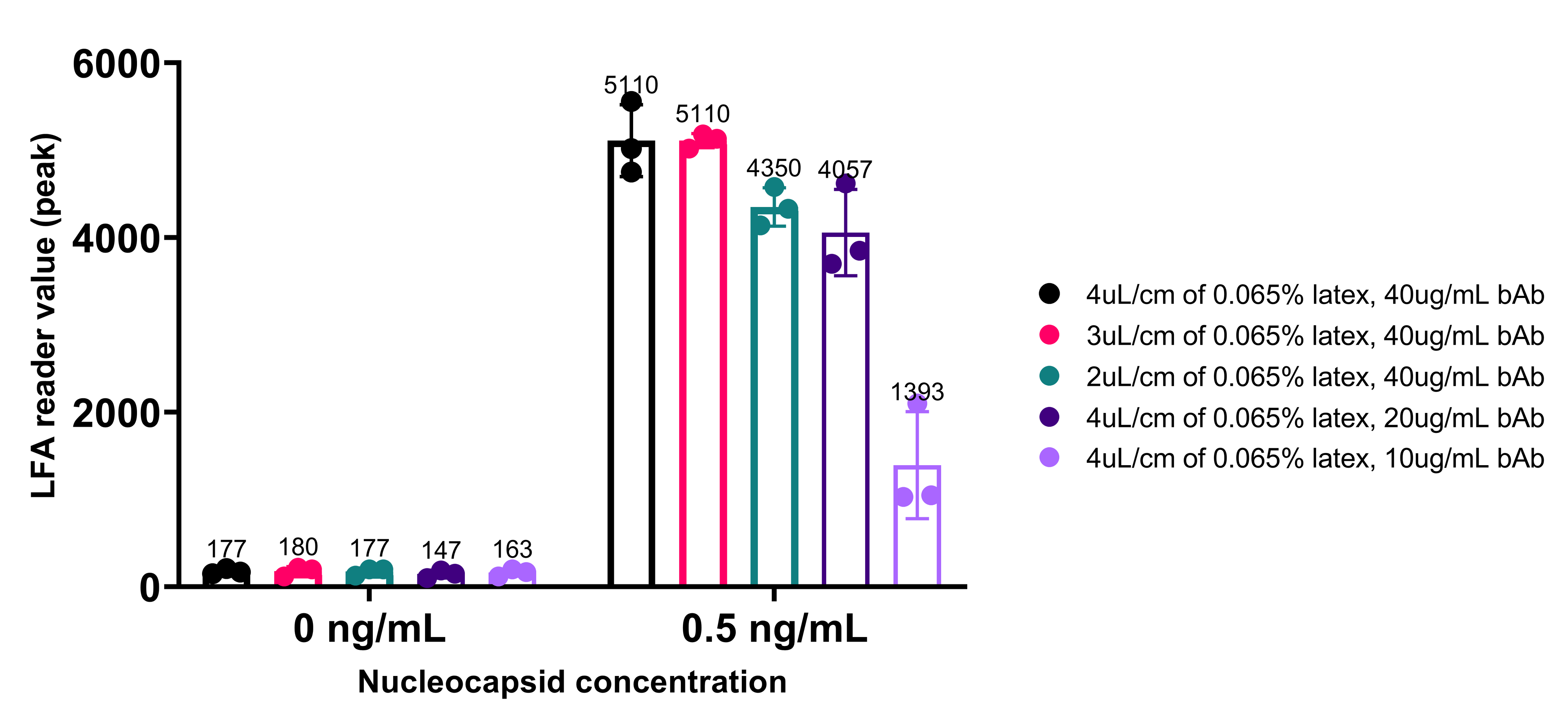

Supplement: S2 Fig — Conjugate pads were sprayed at different rates for the same concentration of latex conjugate, thereby changing the amount of conjugate deposited on each pad. Similar to S1 Fig, there is a steep drop off in signal as biotinylated antibody concentration is decreased. Changes in the speed of deposition for the latex conjugate, however, do not make as significant a difference. (TIF) [file pone.0258819.s003.tif]

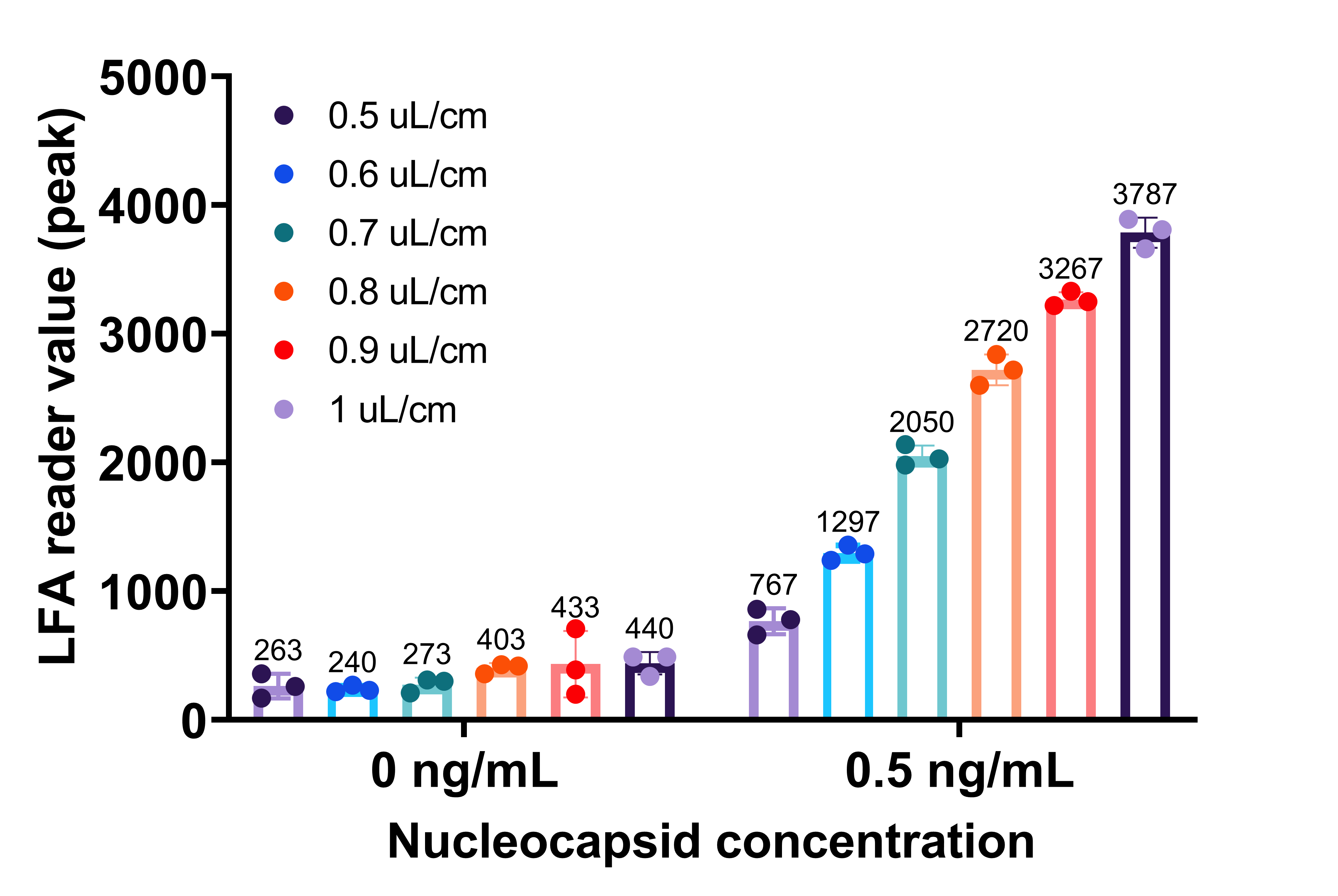

Supplement: S3 Fig — With regards to signal of the negative, the data sets can be grouped into two, with a drop off occurring between 0.8 μL/cm and 0.7 μ L/cm. In our final build, we used 1 μL/cm to maximize positive signal, and found alternate methods to reduce the signal in the negative, including nitrocellulose block and using a more hydrophobic conjugate pad (Lydall 9819). (TIF) [file pone.0258819.s004.tif]

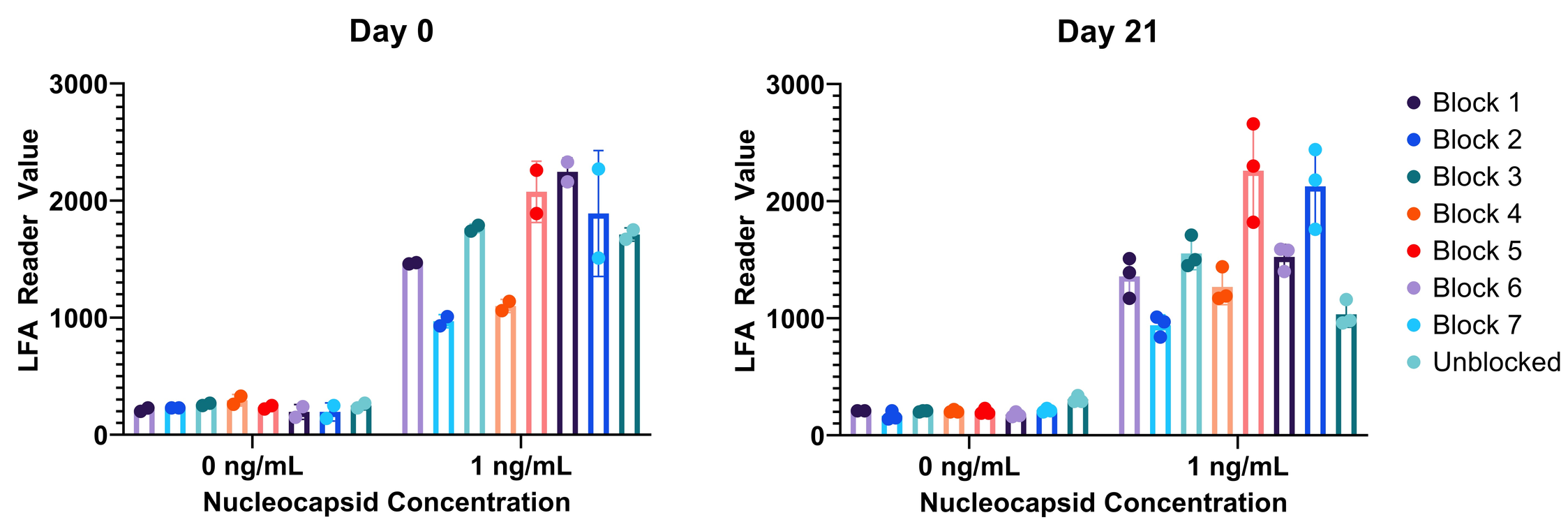

Supplement: S4 Fig — The specifics of each nitrocellulose block can be seen in S1 Table in S1 File. These membranes were stored at 40°C in a sealed mylar bag containing desiccant. Reduced performance is seen in nitrocellulose block 2 and 4 at Day 0. By Day 21, Block 6 and the unblocked condition show the biggest drop off in signal (32.1 and 39.6% respectively). Blocks 5 and 7, both of which contain 2% sucrose and 2% beta lactose with differing amounts of casein and BSA. (TIF) [file pone.0258819.s005.tif]

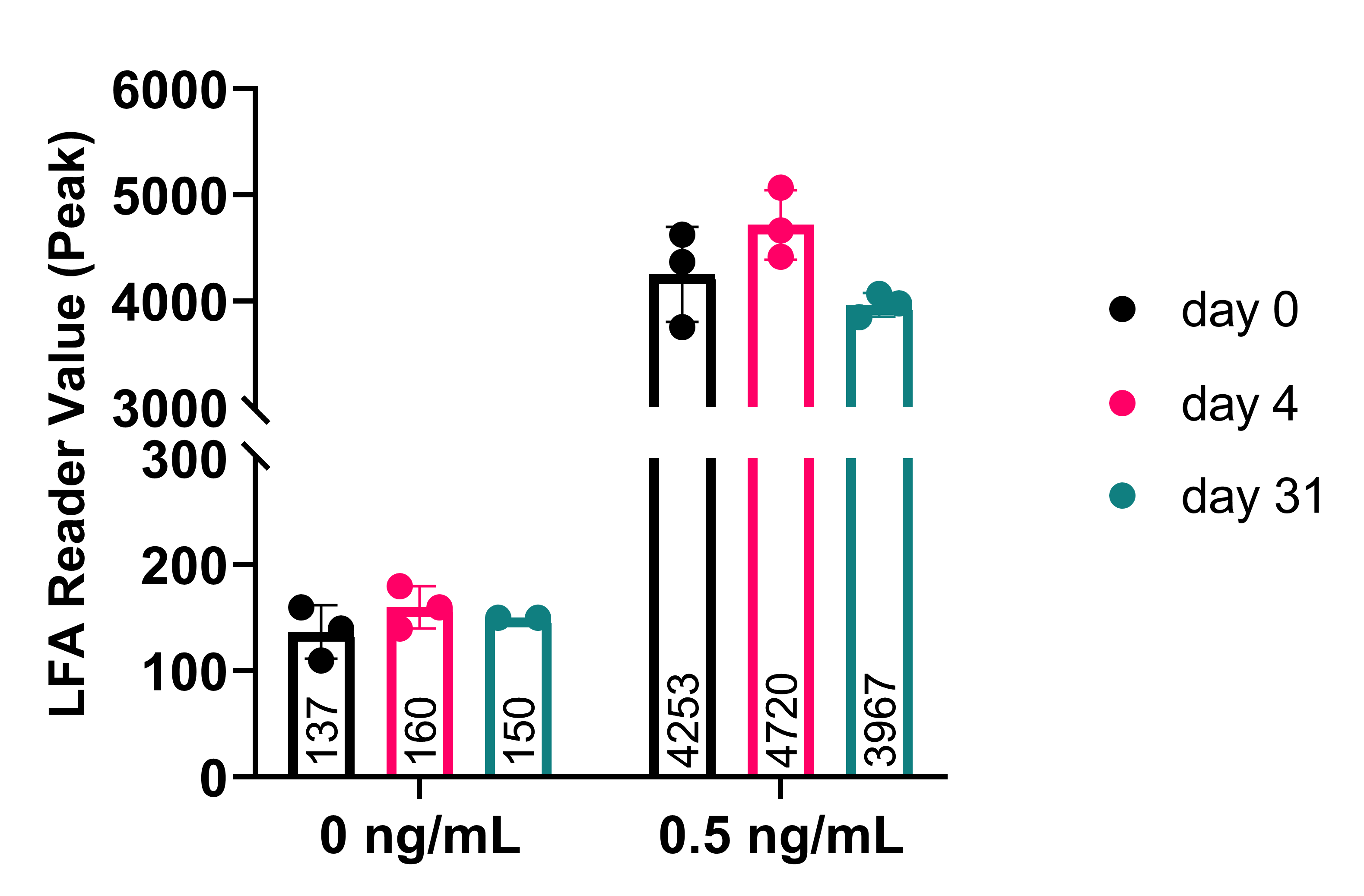

Supplement: S5 Fig — Similar performance was seen when strips were tested at Day 0, Day 4, and Day 31 after being stored at 40°C. (TIF) [file pone.0258819.s006.tif]
