# Supplementary material for: A SARS-CoV-2 coronavirus nucleocapsid protein antigen-detecting lateral flow assay
Source: PLoS One. 2021 Nov 10;16(11):e0258819. doi: 10.1371/journal.pone.0258819 (PMC8580225; doi:10.1371/journal.pone.0258819)
Supplement: Attachment — Submitted filename: response to reveiwers 9_12_2021.docx [file pone.0258819.s007.docx]

Dear Reviewers:

We appreciate the feedback and the opportunity to improve upon our submission. The comments were helpful and helped to improve the quality of our paper.

Several references have change in order to update the manuscript. Previous reference 10 (Alexandersen et al.) was removed as it was an outdated preprint. Previous reference 9 (He et al.) was also removed, as reference 8(Larremore et al.), more directly supports the relevant text (line 43). Reference 2 was updated in order to include the most up to date Covid-19 infection and mortality numbers. References 9, 10, and 11 were added to support the need for additional Covid-19 tests. The clinical performance of the LFA described in this manuscript were recently published. This publication has been added as reference 18.

Below, please see our responses to specific points answered in bold.

Thank you,

Benjamin Grant

**Comments to the Author**

1. Is the manuscript technically sound, and do the data support the conclusions?

The manuscript must describe a technically sound piece of scientific research with data that supports the conclusions. Experiments must have been conducted rigorously, with appropriate controls, replication, and sample sizes. The conclusions must be drawn appropriately based on the data presented.

Reviewer #1: Yes

2. Has the statistical analysis been performed appropriately and rigorously?

Reviewer #1: Yes

3. Have the authors made all data underlying the findings in their manuscript fully available?

The [PLOS Data policy](https://nam12.safelinks.protection.outlook.com/?url=http%3A%2F%2Fwww.plosone.org%2Fstatic%2Fpolicies.action%23sharing&data=04%7C01%7Cben.grant%40ghlabs.org%7C924ec4f8cc0d4bcca64008d94e146ff9%7C5661cfca69e541e1b057d6dedcad082f%7C0%7C0%7C637626672866984404%7CUnknown%7CTWFpbGZsb3d8eyJWIjoiMC4wLjAwMDAiLCJQIjoiV2luMzIiLCJBTiI6Ik1haWwiLCJXVCI6Mn0%3D%7C1000&sdata=iDQRvI%2FKgrJUBIQjv0J8rPpTQFNd%2BbSdNBuxr07l%2BAY%3D&reserved=0) requires authors to make all data underlying the findings described in their manuscript fully available without restriction, with rare exception (please refer to the Data Availability Statement in the manuscript PDF file). The data should be provided as part of the manuscript or its supporting information, or deposited to a public repository. For example, in addition to summary statistics, the data points behind means, medians and variance measures should be available. If there are restrictions on publicly sharing data—e.g. participant privacy or use of data from a third party—those must be specified.

Reviewer #1: Yes

4. Is the manuscript presented in an intelligible fashion and written in standard English?

PLOS ONE does not copyedit accepted manuscripts, so the language in submitted articles must be clear, correct, and unambiguous. Any typographical or grammatical errors should be corrected at revision, so please note any specific errors here.

Reviewer #1: Yes

5. Review Comments to the Author

Please use the space provided to explain your answers to the questions above. You may also include additional comments for the author, including concerns about dual publication, research ethics, or publication ethics. (Please upload your review as an attachment if it exceeds 20,000 characters)

Reviewer #1: A particularly well-written manuscript on a subject important for the diagnosis of Sars-Cov-2 infection. The authors herein format present an open-access visually-read lateral flow assay using commercially-available antibodies and materials for the detection of SARS-CoV-2. The study refers to a previously published paper from the same group in Analytical Chemistry on a related subject. The current work is very technical, very analytical, very precise and rigorously written.

The following few points need to be resolved to improve the manuscript:
1°. The abstract is insufficient and does not reflect the results of the study.

**The abstract has been expanded to better represent the findings of the paper.**
2°. The interest of developing a rapid COVID-19 antigen test with commercial consumables should be discussed for middle and poor countries;

**Additional rationale has been provided for the development of an open-sourced, commercially sourced assay.**

3°. A paragraph on the strengths and limitations of the study must be absolutely written; in particular, it is necessary to insist on the absence of clinical validation of the proposed rapid diagnostic test, on the problem of the choice of the matrices, on the stability of consumables over time depending on the temperature (since positive results in rapid antigenic tests may be possible depending on the temperature).

**We have now published the clinical results and have cited these results in our current manuscript (lines 283-288).**

**We have added a paragraph describing the limitations of this manuscript (lines 319-330). Supplemental figure S5 provides stability data for up to 31 days at 40C, equivalent to approximately 90 days at 25C. We now note this as a limitation (lines 326-330) and note that final stability must be determined by test manufacturers as it can be impacted by production and packaging conditions temperature and humidity.**

4°. A paragraph on the technicality of carrying out such a test must be described (easy or difficult to perform; depending on what level of infrastructure; etc).

**The ease-of-use has now been described in the discussion section [lines 273-276].**

5°. Finally, the economic interest of the manufacture of an open rapid test for COVID-19 antigen diagnosis should be discussed, since many rapid COVID-19 antigenic tests exist, and some are very inexpensive, and even offered in supermarkets.

**We have added the following statement to our manuscript (lines 56-57) “The gap in testing rates between high-income countries and LMICs indicates that well-validated, cost-effective and sufficiently distributed tests are still lacking) The FIND test tracker (reference 10) highlights this fact. We believe that a significant need continues to exist for validated, antigen-based tests with distributed manufacturers.**

6. PLOS authors have the option to publish the peer review history of their article ([what does this mean?](https://nam12.safelinks.protection.outlook.com/?url=https%3A%2F%2Fjournals.plos.org%2Fplosone%2Fs%2Feditorial-and-peer-review-process%23loc-peer-review-history&data=04%7C01%7Cben.grant%40ghlabs.org%7C924ec4f8cc0d4bcca64008d94e146ff9%7C5661cfca69e541e1b057d6dedcad082f%7C0%7C0%7C637626672866994356%7CUnknown%7CTWFpbGZsb3d8eyJWIjoiMC4wLjAwMDAiLCJQIjoiV2luMzIiLCJBTiI6Ik1haWwiLCJXVCI6Mn0%3D%7C1000&sdata=YZgGZlbvFAT8Ey7jf%2BGXTmMChkfs02X1WJhFdRJGWcQ%3D&reserved=0)). If published, this will include your full peer review and any attached files.


If you choose “no”, your identity will remain anonymous but your review may still be made public.


**Do you want your identity to be public for this peer review?** For information about this choice, including consent withdrawal, please see our [Privacy Policy](https://nam12.safelinks.protection.outlook.com/?url=https%3A%2F%2Fwww.plos.org%2Fprivacy-policy&data=04%7C01%7Cben.grant%40ghlabs.org%7C924ec4f8cc0d4bcca64008d94e146ff9%7C5661cfca69e541e1b057d6dedcad082f%7C0%7C0%7C637626672866994356%7CUnknown%7CTWFpbGZsb3d8eyJWIjoiMC4wLjAwMDAiLCJQIjoiV2luMzIiLCJBTiI6Ik1haWwiLCJXVCI6Mn0%3D%7C1000&sdata=DroPoDCRYYeynXr8R5Yv6E2K%2FPWDX0lY6NDQgw%2B%2FJvk%3D&reserved=0).

Reviewer #1: No

While revising your submission, please upload your figure files to the Preflight Analysis and Conversion Engine (PACE) digital diagnostic tool, [https://pacev2.apexcovantage.com/](https://nam12.safelinks.protection.outlook.com/?url=https%3A%2F%2Fpacev2.apexcovantage.com%2F&data=04%7C01%7Cben.grant%40ghlabs.org%7C924ec4f8cc0d4bcca64008d94e146ff9%7C5661cfca69e541e1b057d6dedcad082f%7C0%7C0%7C637626672867004312%7CUnknown%7CTWFpbGZsb3d8eyJWIjoiMC4wLjAwMDAiLCJQIjoiV2luMzIiLCJBTiI6Ik1haWwiLCJXVCI6Mn0%3D%7C1000&sdata=sXgH4YFTYWFQnKAYW3%2Bsp9cH%2BrPYxB5sAVpPYMqLSIA%3D&reserved=0). PACE helps ensure that figures meet PLOS requirements. To use PACE, you must first register as a user. Registration is free. Then, login and navigate to the UPLOAD tab, where you will find detailed instructions on how to use the tool. If you encounter any issues or have any questions when using PACE, please email PLOS at [figures@plos.org](mailto:figures@plos.org). Please note that Supporting Information files do not need this step.
